# Supplementary material for: Analysis of chromatin data supports a role for CD14+ monocytes/macrophages in mediating genetic risk for juvenile idiopathic arthritis
Source: Front Immunol. 2022 Sep 29;13:913555. doi: 10.3389/fimmu.2022.913555 (PMC9559786; doi:10.3389/fimmu.2022.913555)
Supplement: Supplementary file 1 [file Table_1.docx]

**Supplemental Table 1:** Genomic locations of TADs surrounding 28 JIA risk haplotypes

| **Risk Haplotype** | **LD Block Coordinates** | **TAD Coordinates** |
| --- | --- | --- |
| ATP882-IL6R | chr1:154,319,242-154,406,893 | chr1:153,195,001-154,535,008 |
| IL2-21 | chr4:122,151,854-122,619,603 | chr4:120,985,001-122,750,000 |
| ERAP-LNPEP | chr5:96,884,383-97,038,046 | chr5:96,575,001-98,430,000 |
| C5orf56-IRF1 | chr5:132,477,527-132,496,822 | chr5:132,415,000-133,290,000 |
| HLA-DQB1-DRA2 | chr6:32,422,420-32,712,215 | chr6:31,495,002-32,850,000 |
| SH2B3-ATXN2 | chr12:111,395,984-111,645,358 | chr12:111,305,000-112,175,000 |
| ZFP36L1 | chr14:68,784,174-68,794,755 | chr14: 67870001-70260000 |
| TYK2 | chr19:10,317,045-10,381,598 | chr19:10,025,000-10,855,000 |
| UBE2L3 | chr22:21,556,931-21,628,971 | chr22:21,095,001-22,025,000 |
| TIMMDC1-CD80 | chr3:119,406,355-119,529,051 | chr3:118,635,000-120,525,001 |
| JAK1 | chr1:64,924,820-64,975,081 | chr1:64725000-66,250,000 |
| PTH1R | chr3:46,889,988-46,932,682 | chr3:46,440,001-47,855,000 |
| ILDR1-CD86 | chr3:122,023,892-122,102,059 | chr3:121,180,001-123,300,000 |
| AHI1-LINC-00271 | chr6:135,303,673-135,371,709 | chr6:134,450,001-135,750,000 |
| HBP1 | chr7:107,156,092-107,390,877 | chr7:106,800,001-107,885,000 |
| WDFY4 | chr10:48,776,493-48,805,795 | chr10:48,445,001-48,995,000 |
| RNF215 | chr22:30,289,571-30,403,731 | chr22:29,650,001-30,500,000 |
| LTBR | chr12:6,384,185-6,402,830 | chr12:6,055,001-6,685,001 |
| CCR1-CCR3 | chr3:46,141,688-46,420,292 | chr3:45,030,001-46,500,000 |
| PRM1-RM12 | chr16:11,278,799-11,352,790 | chr16:10,995,001-12,065,000 |
| JAZF1 | chr7:28,114,765-28,207,370 | chr7:28,085,001-28,955,000 |
| STAT4 | chr2:191,079,016-191,108,308 | chr2:190,300,001-191,835,001 |
| ANKRD55 | chr5:56,141,024-56,146,422 | chr5:54,440,001-56,610,000 |
| PRR9-LOR | chr1:153,249,128-153,270,022 | chr1:152,025,001-153,490,000 |
| IL2RA | chr10:6,028,313-6,055,320 | chr10:4.850.001-6.465.000 |
| PRRL5 | chr11:36,314,713-36,354,471 | chr11:35420001-36810000 |
| RUNX3 | chr1:24,870,664-24,884,324 | chr1:24,380,001-25,250,000 |
| AFF3-LONRF2 | chr2:100,196,869-100,221,105 | chr2: 99,050,001-101,190,000 |
